# Evaluation of preventive tract embolization with standardized gelatin sponge slurry on chest tube placement rate after CT-guided lung biopsy: a propensity score analysis

**DOI:** 10.1186/s13244-023-01566-8

**Published:** 2023-11-28

**Authors:** Rémi Grange, Mathieu Di Bisceglie, Paul Habert, Noémie Resseguier, Robin Sarkissian, Marjorie Ferre, Michael Dassa, Sylvain Grange, Jean Izaaryene, Gilles Piana

**Affiliations:** 1 Department of Interventional Radiology, University Hospital of Saint-Etienne, University Hospital of Saint-Etienne, Avenue Albert Raimond, 42270 Saint-Priest-en-Jarez, France; 2 Department of Interventional Radiology, Institut Paoli Calmettes, Marseille, France; 3 Department of Imaging, Hospital Nord, Marseille, APHM, Aix Marseille University, Marseille, France; 4 Aix Marseille Univ, LIIE, Marseille, France; 5 Methodological Support Unit for Clinical and Epidemiological Research, University Hospital of Marseille (APHM), Marseille, France; 6 CEReSS- Health Services and Quality of Research, Aix Marseille University, Marseille, France

**Keywords:** Computed tomography, Biopsy, Gelatin sponge, Pneumothorax, Chest tube placement

## Abstract

**Background:**

To evaluate the effect of tract embolization (TE) with gelatin sponge slurries during a percutaneous lung biopsy on chest tube placement and to evaluate the predictive factors of chest tube placement.

**Methods:**

Percutaneous CT-guided lung biopsies performed with (TE) or without (non-TE) tract embolization or between June 2012 and December 2021 at three referral tertiary centers were retrospectively analyzed. The exclusion criteria were mediastinal biopsies, pleural tumors, and tumors adjacent to the pleura without pleural crossing. Variables related to patients, tumors, and procedures were collected. Univariable and multivariable analyses were performed to determine risk factors for chest tube placement. Furthermore, the propensity score matching analysis was adopted to yield a matched cohort.

**Results:**

A total of 1157 procedures in 1157 patients were analyzed, among which 560 (48.4%) were with TE (mean age 66.5 ± 9.2, 584 men). The rates of pneumothorax (44.9% vs. 26.1%, respectively; *p* < 0.001) and chest tube placement (4.8% vs. 2.3%, respectively; *p* < 0.001) were significantly higher in the non-TE group than in the TE group. No non-targeted embolization or systemic air embolism occurred. In the whole population, two protective factors for chest tube placement were found in univariate analysis: TE (OR 0.465 [0.239–0.904], *p* < 0.05) and prone position (OR 0.212 [0.094–0.482], *p* < 0.001). These data were confirmed in multivariate analysis (*p* < 0.001 and *p* < 0.0001 respectively). In the propensity matched cohort, TE reduces significatively the risk of chest tube insertion (OR = 0.44 [0.21–0.87], *p* < 0.05).

**Conclusions:**

The TE technique using standardized gelatin sponge slurry reduces the need for chest tube placement after percutaneous CT-guided lung biopsy.

**Critical relevance statement:**

The tract embolization technique using standardized gelatin sponge slurry reduces the need for chest tube placement after percutaneous CT-guided lung biopsy.

**Key points:**

1. Use of tract embolization with gelatine sponge slurry during percutaneous lung biopsy is safe.

2. Use of tract embolization significantly reduces the risk of chest tube insertion.

3. This is the first multicenter study to show the protective effect of tract embolization on chest tube insertion.

**Graphical Abstract:**

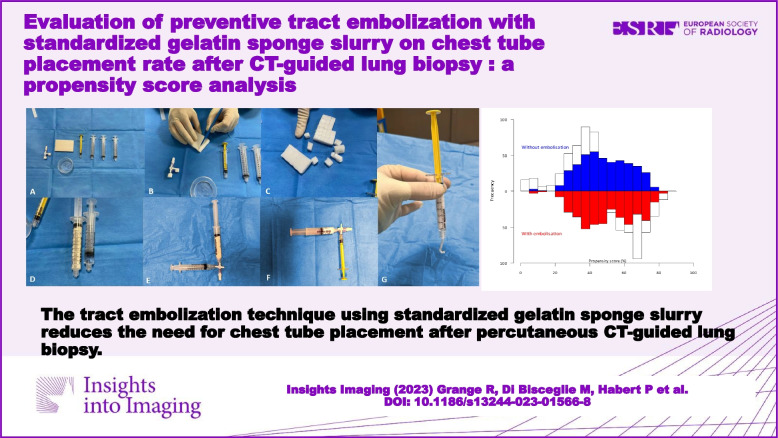

## Background

Percutaneous CT-guided lung biopsy (PCTLB) is a safe and effective procedure for diagnosing suspected malignant nodules and their molecular characteristics for targeted therapies [1], particularly for tumors far from the hilum [2–4]. Pneumothorax is the most common complication of PCTLB, with a rate ranging from 12 to 45% [3, 5]. Pneumothorax is generally asymptomatic; however, chest tube placement can generate discomfort, pain, increased hospital stay, and altered patient quality of life [6]. In the literature, the rate of chest tube placement after PCTLB varies between 2 and 15% [3, 5]. Currently, new mini-invasive bronchoscopy-guided techniques are emerging (electromagnetic navigation, virtual bronchoscopy, radial endobronchial ultrasound, and ultra-thin bronchoscopy) to access peripheral lung tumors. The reported rate of pneumothorax for these techniques is 1.5% (range 0.0–7.5% across studies); of these, 0.6% require chest tube [7]. In a recent meta-analysis, the argument used to promote these techniques despite moderate diagnostic yields (70%) was the invasiveness of PCTLB, citing mainly higher complication rates [8]. Many techniques have been described to decrease the incidence of pneumothorax after PCTLB, some of which involve occlusion of the puncture site during needle removal [9–13]. Some studies have focused on the efficacy and safety of tract embolization (TE) using gelatin sponge slurry [14–16]. The initial results are encouraging; however, the published data were collected from small populations, the rate of pneumothorax and drainage was quite low, the techniques of slurry preparation were not standardized, and the management of pneumothorax, especially the indication for chest tube placement, was not harmonized.

Factors associated to chest tube placement have been highlighted, such as patient age [17, 18], emphysema [19, 20], characteristics of the lesion (location [17, 21, 22], depth [19, 23] size [23]), or characteristics of the procedure (patient position [18, 20], size of coaxial needle [24, 25], angle of needle with the pleura [26], fissural puncture [19]). To date, no large comparative multicenter study has evaluated the predictive factors for chest tube insertion using the TE technique with a standardized gelatinous sponge slurry.

This multicenter retrospective study aimed to evaluate the effect of TE with standardized gelatin sponge slurries during PCTLB on chest tube insertion. The secondary objective was to retrospectively evaluate the predictive factors for chest tube insertion in the entire cohort.

## Materials and methods

### Study design

Institutional Review Board approval (Groupe de Sélection des Projets Cliniques, EMBOPSIE-IPC 2021–088) was obtained to study this retrospective multicenter cohort from three tertiary referral centers. All consecutive PCTLB performed between June 2012 and December 2021 in the first center and between January 2018 and January 2021 in the second and third centers were included. The TE technique was introduced during 2018; prior to this period, no TE was performed, and after this date, the use of this technique remained at the discretion of the operating physicians. The clinical outcome was the occurrence of an in-hospital pneumothorax and the indication for insertion of a chest tube. The outcome was determined at the end of the procedure using computed tomography (CT) scan of the entire lung parenchyma. A retrospective analysis of a multicenter database was performed.

The exclusion criteria were mediastinal biopsies, pleural tumors, and tumors adjacent to the pleura without pleural crossing. Contrary to previous single-center studies, the occurrence of pneumothorax before needle removal was not an exclusion criterion. Biopsies of tumors adjacent to the pleura are known to be less at risk for pneumothorax and chest tube insertion. Making a difference between biopsies with or without the transpleural tract in this location is sometimes difficult. The evaluation of the outcomes of these procedures could be biased, which is why they were excluded.

### PCTLB technique

All procedures were performed under CT guidance (SOMATOM Definition 128 scanner, SOMATOM Confidence, Siemens Healthineers, Erlangen, Germany; Revolution EVO, GE Healthcare, WI, USA; and SOMATOM Definition, Siemens Medical Solutions, Erlangen, Germany). Prior to the procedure, all patients were informed of the risk of and hemoptysis and pneumothorax. All procedures were performed by 10 interventional radiologists with > 5 years of experience. The patients were given oral information on the use of gelatin sponge slurry to reduce the risk of pneumothorax.

First, helical acquisition of the entire lung was performed before the start of the biopsy trajectory planning. Local anesthesia was performed by injecting approximatively 15 mL of 1% lidocaine on the skin, along the path and in contact with the pleura using a 22-G needle. A coaxial needle (an inner stylet and an outer cannula) [27] was then inserted through the skin into the lesion, a semi-automatic needle with a 1 to 2 cm cutting area was used (Temno Evolution Merit Medical, South Jordan, Utah; Quick Core, Cook Medical, Bloomington, IN; Supercore™, Argon Medical Devices, Athens, USA), and the specimen was placed in a formaldehyde tube for analysis. Sequential acquisitions were performed during needle positioning. Breath holding was not required from the patient during the biopsy or when the needle was removed. Immediately after the removal of the coaxial needle, a final acquisition was performed on the entire lung parenchyma to detect pneumothorax or intra-alveolar pulmonary hemorrhage. Following the procedure, patients were monitored at least for a minimum of 4 h in the medical department. In case of dyspnea during monitoring, a new CT scan was performed to ensure that no pneumothorax had appeared or increased.

### TE technique

**Fig. 1:**
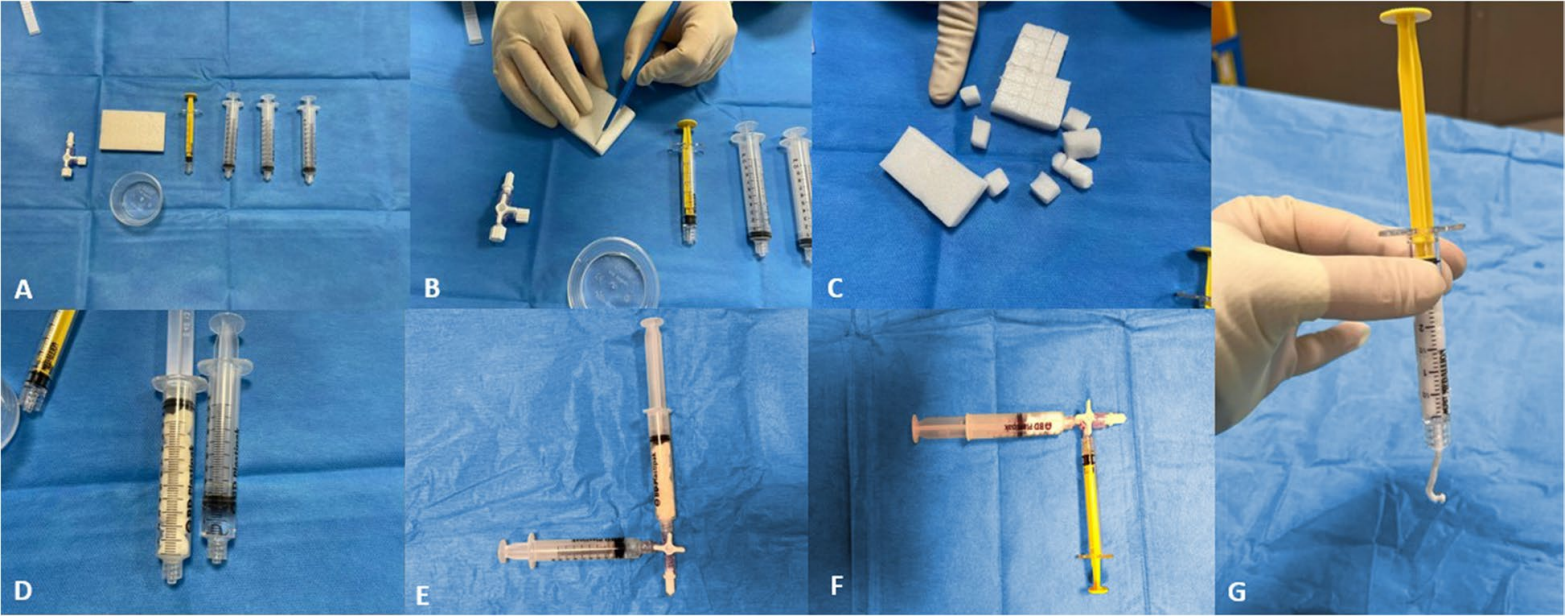
Pictures showing the preparation steps of the gelatin sponge slurry. **a** 3 × 10 mL syringes, 1 × 3 mL syringe, 1 × gelatine sponge plate, 1 × 3-way stopcock, 1 × iodine cup. **b** One gelatine sponge plate is cut with a scalpel. **c** 2/3 of the gelatine sponge plate is cut into 5 × 5 mm pieces. **d** The pieces of gelatine sponge are put into a 10-mL syringe and the iodine into a 2-mL syringe. **e** The two syringes are mixed using the 3-way stopcock. **f** The mixture is loaded into a 3-mL syringe. **g** The syringe is tested for cohesion

Gelatin sponge slurry preparation was standardized at the three centers (supplemental data). Two thirds of the single gelatin sponge plate (Gelitaspon, Gelita Medical, Amsterdam, The Netherlands) was cut into small pieces (5 × 5 mm), loaded into a 10-mL syringe, and mixed with no more than 2 mL of iodinated contrast agent (Iopamiron, Guerbet) with a three-way stopcock to obtain a compact and dense texture. Then, the mixture was loaded into a 3-mL syringe. To test the quality of the preparation before each use, the mixture was pushed out of the syringe. If the mixture was sufficiently dense and cohesive to adhere to the syringe, the product was considered satisfactory and could be used (Fig. 1). If the mixture did not stick to the syringe, it was not validated for use and discarded, and a fresh mixture was prepared. Once the biopsy was completed, the gelatin sponge slurry was injected into the needle channel until resistance to the injection was felt (less than 1 mL). The needle trocar was then inserted at the base of the coaxial needle, and the coaxial needle was withdrawn while holding the trocar so that the gelatin sponge slurry replaced the needle tract. No other maneuvers were performed to reduce the risk of pneumothorax. The final helical CT scan also verified the absence of non-target embolization or systemic air embolism.

### Data collection

Variables related to the tumors were retrospectively collected from the initial procedure images (fissural tract, lesion size, patient position, needle-pleural angle, distance to the pleura, emphysema) in each center. Variables related to the patients were retrospectively collected from the computerized patient file (sex, age, history of thoracic surgery). Variables related to the procedures (time of procedure, pneumothorax, chest tube insertion, size of coaxial/needle) were retrospectively collected from the biopsy report in each center.

Puncture time was defined as the time between the first CT a with the co-axial needle in the lung and the final acquisition. The distance to the pleura was defined as the distance from the pleural puncture to the target lesion along the biopsy-track axis. Emphysema along the needle tract was evaluated qualitatively according to the Fleischner Society classification [26] and was assigned as no significant emphysema (group a) or significant emphysema (groups b to e). The needle-pleural angle was defined as the angle (°) between the line defined by the needle and the line perpendicular to the pleural tangent. A diagnosis of immediate pneumothorax was established if any air density was visible in the pleural space on the final chest CT scan. Systemic gas embolism was defined as the presence of gas in the arterial or venous circulatory system on the final acquisition. Non-target embolization was defined as the presence of an iodinated opacity within the vascular system and adjacent bronchi peripheral to the sampling area. The indication to insert a chest drain was standardized in the three centers and based on the recommendations of the Pleural Disease Guidelines 2010 established by the British Thoracic Society, according to the volume of pneumothorax and/or clinical symptoms [28]. No intervention was performed for patients without pneumothorax or for patients with a small pneumothorax (distance < 2 cm between the parietal and visceral pleura at the level of the hilum). Exsufflation was performed in asymptomatic patients with large pneumothorax. A chest tube was positioned for patients with exsufflation failure, large pneumothorax (distance > 2 cm between the parietal and visceral pleura at the level of the hilum), respiratory symptoms, or increased pneumothorax on the CT scan performed during monitoring. A chest radiograph 4 h after the procedure was not routinely performed in the three centers.

### Statistical methods

Results are presented as mean ± standard deviation (SD) or median [Q1-Q3], according to the distribution for continuous variables and as number and frequency for categorical variables. Categorical variables were compared using the chi-square test, and continuous variables were compared using Student’s *t*-test. If a variable had less than 5% missing data, we performed a median imputation for quantitative variables and a mode imputation for qualitative variables. If a variable had between 5 and 20% missing data, a multiple chain equation multiple imputation (MICE) was performed. The predictive factors for chest tube insertion were assessed using univariable and multivariable analyses. Odds ratios (OR) and 95% confidence intervals are reported as appropriate. For the multivariable analysis, logistic regression was performed, and we selected the candidate variables from the set of collected variables in such a way that less than 20% of patients had missing data or variables with less than 5% missing values. The predictor variables TE, emphysema, tract length, and prone position were introduced in the multivariable model based on *p* < 0.2 in the univariable analysis and according to a priori data from the literature. Statistical significance was set at *p* < 0.05. All statistical analyses were conducted using the R +  + software.

Propensity score-based matching was developed using a logistic regression model that included 8 variables (age, lesion size, tract length, sex, prone position, fissural tract, emphysema, needle size) and/or to the outcome (chest tube insertion), regardless of their statistical significance using a non-parsimonious approach. Using the propensity score, non-TE patients were matched to TE patients. A nearest-neighbor 1:1 matching algorithm was applied, with a caliper width of 0.2 SD of the logit of the propensity score. Standardized difference before and after matching were estimated (with their 95% confidence intervals) to assess the quality of the propensity score matching procedure. A mirrored histogram of distribution of propensity scores for non-TE (bars above the zero line) versus TE (bars below the zero line) was plotted. The R package MatchIt was used for the propensity score-based matching.

## Results

### Population

**Fig. 2:**
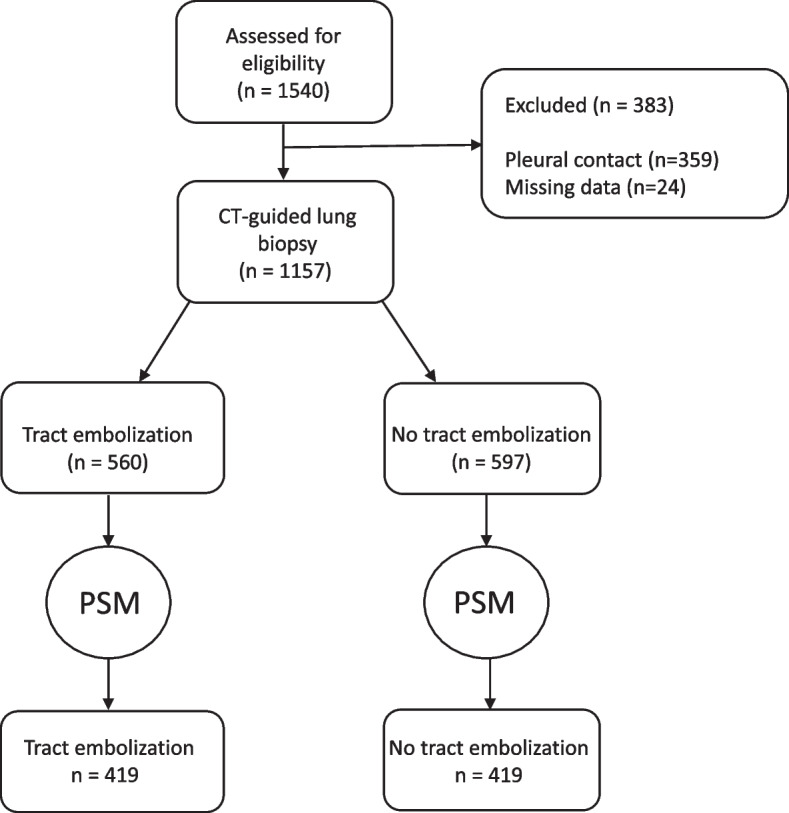
Study flow diagram of the study

The study participants’ repartitions are detailed in the flowchart (Fig. 2). A total of 1157 procedures were included, among which 597 (52%) were without TE and 560 (48%) were with TE.

The characteristics of TE and non-TE groups were not similar. Table 1 shows the number of patients in each group and the number of data points for each variable of interest. The patients in the TE group had longer trajectories (*p* < 0.001), smaller lesion sizes (*p* < 0.01), more emphysema (*p* < 0.001), puncture trajectories that more often passed through the fissures (*p* < 0.05), and larger needles (*p* < 0.05).

**Table 1:** Characteristics of TE and non-TE groups before and after propensity score matching

	Before matching				After matching			
	Control group (*n* = 597)	Embolization group(*n* = 560)	*P*	SMD	Control group (*n* = 419)	Embolization group (*n* = 419)	*P*	SMD
Age, mean ± SD	66.6 ± 9.3	66.4 ± 9.1	0.77	0.017	66.7 ± 9.1	66.2 ± 8.8	0.407	0.05
Tract length (mm) mean (± SD) [Q25–75]	22.9 ± 11.4 [13–33]	26.1 ± 2.6	< 0.001	0.206	24.3 ± 11.5	24.8 ± 1.9	0.581	0.035
Tumor size (mm) mean (± SD) [Q25–75]	28.2 ± 14.4 [13–35]	24.8 ± 14.0	< 0.01	0.178	26.6 ± 13.5	25.6 ± 14.7	0.473	0.053
Emphysema *n*, (%)	203 (34.0)	374 (66.8)	< 0.001	0.699	174 (41.6)	191 (45.8)	0.224	0.077
Prone position, *n* (%)	264 (44.2)	281 (50.2)	< 0.01	0.235	192 (45.6)	193 (46.3)	0.844	0.014
Fissural tract, *n* (%)	8 (1.3)	20 (3.6)	< 0.05	0.26	8 (1.9)	12 (2.9)	0.363	0.063
Sex, *n* (%)	324 (54.3)	260 (46.4)			216 (51.3)	193 (46.3)	0.407	
Needle diameter, *n* (%)			< 0.05	0.482				0
20G	39 (6.5)	4 (0.7)			0 (0)	3 (0.7)	0.997	
19G	9 (1.5)	0 (0)			0 (0)	0 (0)		
18G	549 (91.9)	556 (99.3)			419 (0)	416 (99.3)		
Pneumothorax, *n* (%)	268 (44.9)	146 (26.1)	< 0.001		186 (44.5)	113 (27.1)	< 0.001	
Chest tube, *n* (%)	29 (4.8)	13 (2.3)	< 0.001		25 (6.0)	11 (2.6)	0.017	

*PSM* propensity score matching, *TE* tract embolization

### Outcomes

In the entire cohort, 414 (35.8%) patients had pneumothorax, and 42 (3.6%) patients required chest tube insertion after PCTLB. In the non-TE group, 268 (44.9%) patients had pneumothorax, and 29 (4.8%) required chest tube insertion. In the TE group, despite initial characteristics showing a population at higher risk for chest tube insertion, 146 (26.1%) had pneumothorax (*p* < 0.001), and 13 (2.3%) required chest tube insertion (*p* < 0.001). No nontarget embolization or systemic air embolism was observed in the TE group (Table 2). One systemic air embolism was observed in the non-TE group. No pneumothorax was detected during the monitoring if it was not initially present. No patient was readmitted to hospital for delayed pneumothorax, and no patient was readmitted for chest tube insertion.

**Table 2:** Factors affecting chest tube placement in the study population in univariable and multivariable analysis. A *p *value < 0.05 was considered statistically significant

**Characteristic**				**Univariate analysis**		**Multivariate analysis**	
		**No chest tube (** ***n*** ** = 1115)**	**Chest tube (** ***n*** ** = 42)**	**OR (95% CI)**	***p value***	**OR (95% CI)**	***p value***
Age, mean (± SD)		66.6 ± 9.2	65.2 ± 8.2	0.991 (0.967–1.016)	0.503	-	-
Tract length (mm), mean (± SD) [Q25–75]		24.1 ± 11.8 [13.0; 33.0]	34 ± 16.2 [21; 45.6]	1.034 (1.017–1.051)	< 0.0001	1.0364 (1.018–1.055)	< 0.0001
Tumor size (mm), mean (± SD) [Q25–75]		26.6 ± 14.4 [13.0; 35.0]	24.9 ± 12.2 [13.0; 36.8]	0.994 (0.977–1.012)	0.549	-	-
History of lung surgery, *n* (%)		23 (3.0%)	0 (0%)	0.000 (0–∞)	1	-	-
Tract embolization, *n* (%)		547 (49%)	13 (31%)	0.465 (0.239–0.904)	0.023	0.311 (0.148–0.632)	< 0.001
Emphysema, *n* (%)		303 (27%)	32 (77%)	35.71 (1.916–6.711)	< 0.0001	4.237 (2.155–8.333)	< 0.0001
Prone position, *n* (%)		545 (49%)	7 (17%)	0.212 (0.094–0.482)	< 0.001	0.225 (0.097–0.521)	< 0.0001
Fissural tract, *n* (%)		24 (2.0%)	4 (10%)	4.886 (1.615–14.778)	< 0.01	3.836 (1.139–12.928)	< 0.05
Sex, *n* (%)	Female	558 (50%)	15 (36%)	1.813 (0.954–3.444)	0.075	1.112 (0.557–2.221)	0.762
	Male	557 (50%)	27 (64%)				
Needle diameter *n* (%)	18 G	1066 (95%)	39 (93%)	-	-	-	-
	19 G	9 (1%)	0 (0%)	1.702 (0.719–4.033)	0.226		
	20 G	40 (4%)	3 (7.7%)	66427.6 (0–∞)	0.986		

### Factors affecting chest tube insertion

 In the whole population and univariable analysis, track length (*p* < 0.001), emphysema (*p* < 0.0001), and fissural tract (*p* < 0.01) were associated with chest tube insertion. Prone position (*p* < 0.001) and TE (*p* < 0.05) had a protective effect on chest tube insertion. The multivariable analysis confirmed these results. Two protective factors for chest tube insertion were identified: TE (OR 0.374 [0.17; 0.73], *p* < 0.001) and prone position (OR 0.25 [0.1; 0.5], *p* < 0.0001) (Table 2)).

### Propensity score analysis

**Fig. 3:**
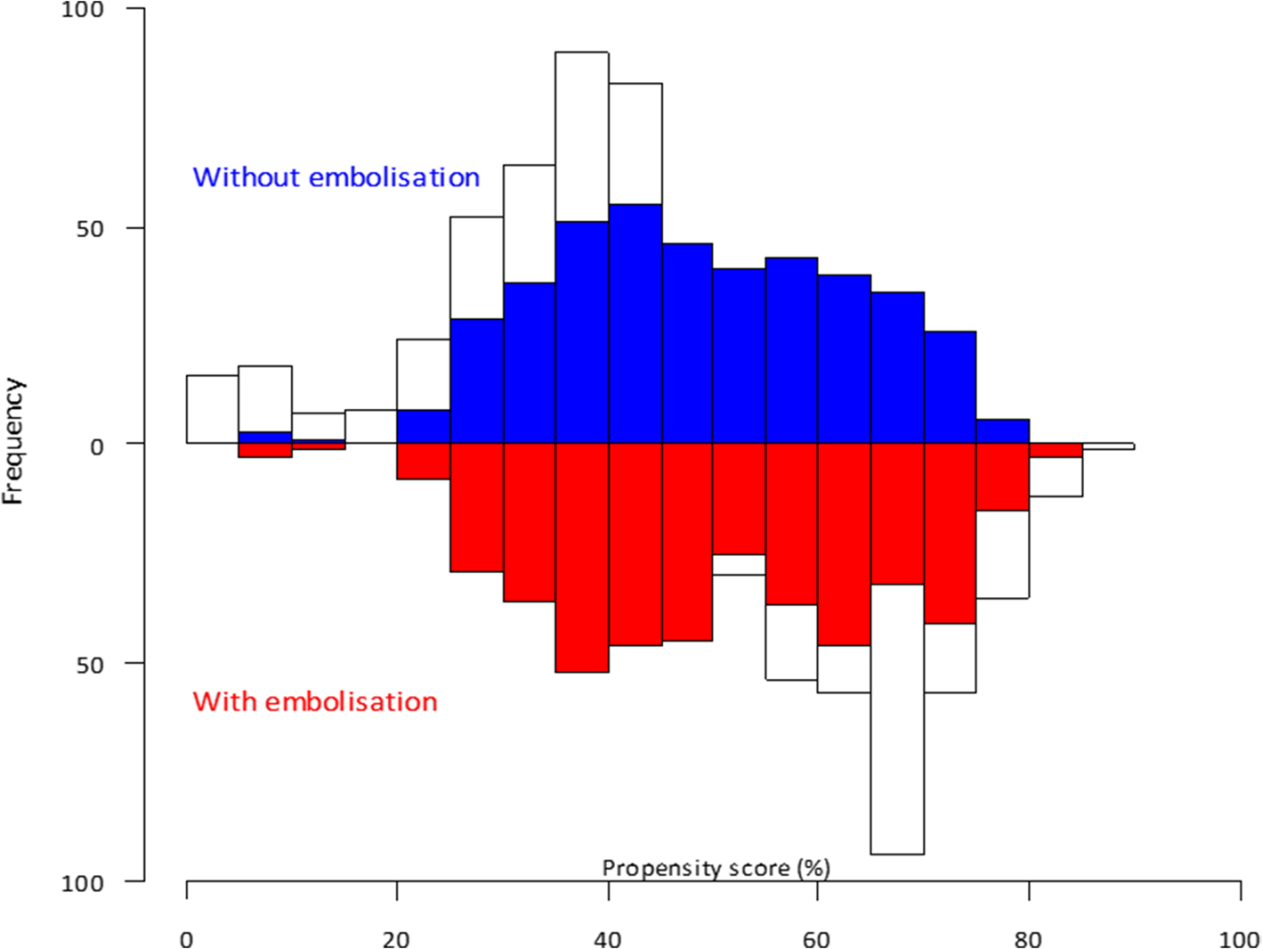
Propensity score distribution of TE group (red) and non-TE group (blue). TE, tract embolization

To further reduce the confounding of other factors between TE embolization and non-TE group, a 1:1 PSM analysis was performed. Age, lesion size, tract length, sex, prone position, fissural tract, emphysema, and needle size were matched. As a result, 419 patients with TE embolization and 419 patients without TE embolization were reserved. After matching, the baseline and main characteristics were comparable (*p* > 0.05 and SMD < 0.2) (Fig. 3 and Table 1). In the matched cohort, 113 (27.1%) patients had pneumothorax in the TE group and 186 (44.5%) in the non-TE group (*p* < 0.001), resulting in 11 (2.6%) chest tube insertion in the TE group and 25 (6.0%) in the non-TE-group (*p* = 0.017).

Compared to the non-TE group, we found that TE reduces significatively the risk of chest tube insertion (OR = 0.44 [0.21–0.87], *p* < 0.05) after propensity score matching (Table 3).

**Table 3:** Effect of Tract embolization on chest tube placement after propensity score matching. A *p *value < 0.05 was considered statistically significant

*n* = 838		OR	*p *value
Tract embolization	0	1	-
	1	0.44 [0.21–0.87]	0.0172

## Discussion

Based on a large population, with a sufficient number of pneumothorax requiring pleural drainage, managed in a harmonized approach following international recommendations, TE with a standardized gelatin slurry allows a significant reduction in chest tube insertion (4.8% vs. 2.3%) after PCTLB.

Technological developments in the field of endoscopic biopsy, especially with cone-beam CT guidance [29], are gaining importance. The advantages of PCTLB are the ability to obtain larger samples and greater availability. Compared to previous techniques, interventional radiologists must prioritize low complication rates and patient comfort. The indication for chest tube insertion varies according to the operator, explaining the variability in chest tube placement rates in the literature [2, 30]. The Society of Interventional Radiology recommends a pneumothorax rate of 45% or less and a chest tube insertion rate of 20% or less. The British Thoracic Society has more restrictive recommendations, with a pneumothorax rate of 20.5% or less and a chest tube insertion of 3.1% or less [28]. A systematic review of the literature including 23 104 patients showed a drainage rate of 6.9%. Many tools have been tested to reduce the rates of pneumothorax during needle removal: TE using autologous blood patch [9, 10, 31], saline [32], hydrogel plugs [11], biopsy site-down technique, and roll-over to puncture site-down maneuver [33, 34]. Even if TE with saline showed the most impressive results, the rate of drainage in the non-TE group was unusually low, especially with the use of 16G needles (0.8% drainage) [32]. In the study by Li et al. [32], only patients with symptomatic pneumothorax were drained (eight patients had moderate pneumothorax and six patients had abundant pneumothorax on CT, however, only 10 drainages). These factors raise the problem of standardization of pneumothorax management in interventional radiology. On the one hand, TE with a plug and blood patch showed a significant reduction in pneumothorax and drainage rates. On the other hand, rollover (puncture site down), deep expiration, and breath-hold on needle extraction showed only trends but no significant protective effect, highlighting embolization compared to other techniques. Among the prospects to be considered, the development and marketing of ready-to-use slurries could encourage operators to adopt this technique. Randomized studies could help determine which embolization agent is more effective for this indication (serum, gelatin, or blood). Other “salvage” techniques could be associated with failure of this procedure in patients and in particular. Recommendations in terms of management of pneumothorax induced by interventional radiology procedures could evolve, notably on the indication of drainage and the use of embolization techniques, thanks to the publication of high-level evidence studies.

Unlike other studies, we included patients with pneumothorax that occurred before needle removal. The proportion of this subpopulation was not counted but the analysis was done on an intention-to-treat basis. Indeed, even if the pneumothorax occurred before the removal of the needle, once the tumor was punctured and the biopsy completed, the operators who chose to embolize the pathway before the occurrence of the pneumothorax tried to embolize the pathway in all cases. The occurrence of pneumothorax before needle removal never had any consequence on the a priori decision to embolize the pathway or not. Excluding these patients may lead to underestimation of the protective effect of gelatin sponge slurry on the occurrence of pneumothorax.

Concerns associated with the use of gelatin sponges can be expressed. As air embolism can occur during a lung biopsy, it is possible that the embolization agent injected into the tract may result in a venous or arterial embolism. There are several reasons why we think that this risk is theoretical. First, all previous studies have demonstrated the safety of TE techniques, especially using gelatin sponges. Then, pulmonary vessels are commonly avoided during biopsy needle insertion; if this is not the case, slurries are far more viscous than air, and the amount of gelatin sponge injected through the coaxial needle is small. Third, the use of a gelatin sponge in hemostasis embolization demonstrates that the effects of embolization are not permanent. For all these reasons, we believe that the theoretical risk is largely compensated for the beneficial effect of embolization. However, in view of the heterogeneity of gelatin preparation methods in different studies and the theoretical risk of nontarget embolization, some precautions have been taken. We carried out preliminary tests for safety considerations to obtain the thickest possible material, and the injection was performed only to fill the lumen of the needle removed with a fixed-point technique. An iodinated contrast medium was used to assess the extent of embolization.

Our study had several limitations. First, this is a retrospective study with inherent limitations. Chronologically, the two groups, TE and no TE, were successive and over a long period, similar to the two previous retrospective studies [14, 35]. The learning curve of the radiologists could overestimate the difference in pneumothorax rates between the two groups, as the use of gelatin sponge slurry is a recent routine practice in the institute where the study was conducted. The present study did not systematically report the occurrence of alveolar hemorrhage or hemoptysis. However, previous studies have shown no impact of TE with a gelatin sponge slurry on the rate of hemoptysis [15]. The diagnostic yields were not assessed.

In conclusion, based on a large population with a sufficient number of pneumothorax requiring pleural drainage, managed in a harmonized approach following the international recommendations, TE with a standardized gelatin slurry allows a significant reduction in chest tube insertion after PCTLB.

## Data Availability

Data sharing is not applicable to this article as no datasets were generated or analyzed during the current study.

## Abbreviations

CT: Computed tomography
OR: Odds ratios
PCTLB: Percutaneous computed-tomography-guided lung biopsy
TE: Tract embolization
